# DDX3X mRNA Expression in T Cells Is Associated with the Severity and Mortality of Septic Patients

**DOI:** 10.1155/2022/5176915

**Published:** 2022-02-08

**Authors:** Yukun Liu, Yongsheng Zhang, Qinxin Liu, Weiming Xie, Xiang Wang, Fan Yang, Wei Gao, Xiangjun Bai, Zhanfei Li, Yuchang Wang

**Affiliations:** ^1^Department of Plastic Surgery, Tongji Hospital, Tongji Hospital of Tongji Medical College, Huazhong University of Science and Technology, Wuhan 430030, China; ^2^Trauma Center/Department of Emergency and Traumatic Surgery, Tongji Hospital of Tongji Medical College, Huazhong University of Science and Technology, Wuhan 430030, China

## Abstract

**Purpose:**

DDX3X acts as the critical checkpoint of death in stressed cells. The purpose of this study was to evaluate the mRNA expression level of DDX3X in T cells in peripheral blood of patients with sepsis and to explore its correlation with the prognosis of sepsis.

**Methods:**

Seventy-nine patients with traumatic sepsis were enrolled in this prospective cohort study. Blood samples were collected within 24 hours after the diagnosis of sepsis or septic shock, and the mRNA expression level of DDX3X in T cells was detected by PCR.

**Results:**

The level of DDX3X mRNA in T cells was significantly increased in septic patients as well as in septic shock patients. The level of DDX3X mRNA was negatively correlated with T cell count and positively correlated with acute physiological and chronic health assessment (APACHE) score and sequential organ failure assessment (SOFA) score (*P* < 0.01). The area under the curve (AUC) of the receiver operating characteristic (ROC) curve was 0.79 (95% confidence interval (CI), 0.68-0.90). A Cox proportional hazard model identified an association between an increased DDX3X mRNA level (≥1.575) and the risk of 28-day mortality (hazard ratio = 9.540, 95% CI, 2.452-37.108).

**Conclusions:**

High level of DDX3X mRNA in T cells in sepsis is associated with the severity of sepsis and the mortality of patients with sepsis.

## 1. Introduction

Sepsis is a life-threatening organ dysfunction caused by an uncontrolled response to infection in hosts and is the leading cause of death in critically ill patients [1]. Sepsis is recognized to be involved in the early activation of both pro- and anti-inflammatory responses, along with alterations in other immunologic and nonimmunologic pathways [2]. Immunosuppression or immune paralysis is considered to be the main reason of long-term complications and death from sepsis [3]. Therefore, it is of particular importance to explore the potential mechanism of immune homeostasis disorder for the prevention and treatment strategy of sepsis.

Lymphocytopenia and immune dysfunction are fundamental characteristics of immunosuppression [3]. A decrease in the number of immune cells, especially lymphocytes, is the first sign of immune paralysis [4]. A previous study has found that lymphocytopenia and immunosuppression in patients with advanced sepsis were associated with CD4+ and CD8+ T cell apoptosis [3]. In addition, sepsis is able to impair their proliferative and effector functions to antigen-mediated stimuli [5]. Persistent immune imbalance leads some sepsis survivors to present with persistent inflammation, immunosuppression, and catabolic syndrome (PICS), characterized by a significant decrease in T lymphocytes [6, 7]. The design that targeted immunoregulatory therapy based on T cells is considered to be a potential measurement to improve the prognosis of sepsis.

As a member of the highly conserved DEAD-box RNA helicase family, Ddx3x has homologous genes in various eukaryotes [8]. DDX3X is involved in the regulation of several physiological functions, including transcription, RNA cleavage and modification, translation initiation, and apoptosis [9]. Current explorations on DDX3X mainly focus on its regulatory role in HIV and HCV infection. In addition, DDX3X modulates the proliferation and activity of tumor cells, such as lung cancer [10], colon cancer, and breast cancer [11]. Several studies have shown that high levels of DDX3X in p53 cells can broadly activate external apoptotic pathways after DNA damage [12]. Knockdown of DDX3X can reduce the level of p53, while overexpression of DDX3X can increase the level of p53 after DNA damage. DDX3X is obviously involved in the regulation of p53 accumulation after DNA damage in cells containing functional p53 through caspase activation [12]. Based on these findings, we speculate that DDX3X may be involved in lymphocyte depletion. Accordingly, in this study, we investigated the expression of DDX3X on T cells in peripheral blood of patients with sepsis and evaluated its clinical significance in sepsis.

## 2. Methods

### 2.1. Research Setting and Study Participants

As a prospective cohort study, patients and samples were collected from the Trauma Center/Emergency Trauma Surgery Department of Tongji Hospital, Tongji Medical College, Huazhong University of Science and Technology. Sepsis and septic shock patients were included according to sepsis-3 [1]. Clinical data and peripheral blood samples were obtained with informed consent. Septic shock was identified as a clinical construct of sepsis with persisting hypotension requiring vasopressors to maintain mean arterial pressure (MAP) ≥ 65 mmHg and having a serum lactate level > 2 mmol/L despite adequate volume resuscitation [1]. All septic patients were treated according to the guidelines of the Sepsis Campaign for Survival [13]. Exclusion criteria included autoimmune disease, inherited or acquired immune deficiency, long-term use of immunosuppressants, acute myocardial infarction, or thromboembolic events. The scheme was approved by the Medical Ethics Committee of Tongji Hospital, Tongji Medical College, Huazhong University of Science and Technology. All procedures are carried out in accordance with relevant guidelines and regulations.

### 2.2. Clinical Data Collection

Clinical characteristics were collected, including demographic characteristics, vital signs, past medical history, laboratory tests, radiographic findings, diagnosis, and outcomes. SOFA and APACHE II scores were calculated and collected within 24 hours of diagnosis of sepsis.

### 2.3. Collection of Blood Samples and T Cells

Venous blood samples were collected with EDTA vacuum tubes within 24 hours of diagnosis of sepsis or septic patients. The Ficoll-Hypaque density gradient centrifugation method (TBD Science; Tianjin, China) is according to the manufacturer's instructions. T cells were extracted from peripheral blood by magnetic bead sorting and counted.

### 2.4. RNA Isolation and Quantitative RT-PCR (qRT-PCR)

Total RNA was extracted from T cells by using Trizol, and reverse-transcribed to cDNA by using the qScript cDNA Synthesis Kit (Quantabio). Quantitative real-time PCR was performed for analysis of gene expression by using SYBR Green Gene Expression Assays (Bio-Rad). The relative quantitative gene expression against an internal control, GAPDH was performed using the 2^−*ΔΔ*Ct^ method. The primer pairs used were as follows: DDX3X forward, 5′-ATGGCTTGTGCCCAAACAG-3′ and reverse, 5′-CGCCTGGACCATCTGAATAAA-3′ [14]; GAPDH forward, 5′-CTTCATTGACCTCAACTAC-3′ and reverse, 5′-GCCATCCACAGTCTTCTG-3′.

### 2.5. Statistical Analysis

Data are described as percentages or median (95% confidence intervals). The classified data were analyzed by the chi-square test or Fisher's exact test. Continuous variables were analyzed using Student's *t*-test or ANOVA. The correlation between DDX3X mRNA and APACHE II or SOFA score was assessed by Pearson correlation analysis. Receiver operating characteristic curve (ROC) was established and area under the ROC curve (AUCs) was determined to evaluate the predictive quality of DDX3X mRNA. The 28-day survival and nonsurvival groups were analyzed using Cox proportional hazard models, which predicted 28-day mortality by ROC based on percentages of DDX3X mRNA cutoff values. The survival curve was analyzed by the Kaplan-Meier method and log-rank test. Statistical analysis was performed using GraphPad Prism 5.01 (GraphPad Software, Inc., La Jolla, CA, USA) or IBM SPSS Version 23 (IBM Corp., Armonk, New York, USA). *P* < 0.05 was considered statistically significant.

## 3. Results

### 3.1. Demographic Characteristics of the Overall Study Population

According to the inclusion and exclusion criteria, a total of 85 patients were included. Four patients were excluded due to immune disease, and two patients were removed without blood samples within 24 hours of the diagnosis of sepsis. A total of 52 sepsis and 27 septic shock patients were enrolled in this study. During the follow-up period, 17 (21.52%) patients died within 28 days. The most common site of infection is the lungs (78.48%), followed by the abdomen (7.59%).

### 3.2. Expression of DDX3X mRNA in T Cells of Sepsis

**Figure 1:**
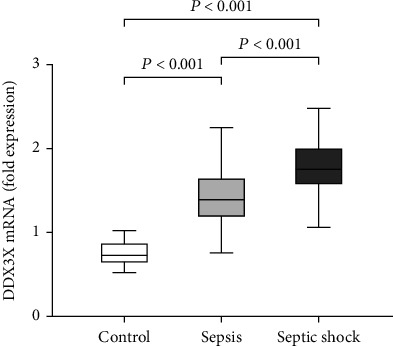
The expression level of DDX3X mRNA on T cells. The expression of DDX3X mRNA in sepsis patients and septic shock patients was higher than the healthy volunteer group (1.43 ± 0.37, 1.75 ± 0.34 vs. 0.75 ± 0.16, *P* < 0.001); besides, the expression level of DDX3X mRNA in septic shock patients was higher than that in the sepsis patients (1.75 ± 0.34 vs. 1.43 ± 0.37, *P* < 0.001).

Patients were divided into two groups: sepsis and septic shock. Ten age- and sex-matched healthy volunteers were served as controls, and no clinical evidence of infection was confirmed by physical examination. There was no significant difference in age and sex between sepsis and septic shock patients. APACHE II, SOFA scores, and procalcitonin levels were higher in the septic shock group than those in septic patients (Table 1). DDX3X mRNA expression in patients with sepsis and septic shock was significantly higher than that in the control group (Figure 1). In addition, results showed that DDX3X mRNA level was significantly higher in the septic shock group than that in the sepsis group (Figure 1).

### 3.3. DDX3X mRNA Was Associated with Severity of Sepsis

**Figure 2:**
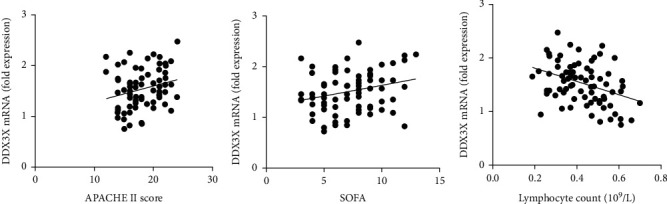
Positive correlations were observed between the plasma DDX3X mRNA level and the (a) APACHE II and (b) SOFA scores. However, DDX3X was significantly negatively correlated with (c) T lymphocyte count.

Pearson correlation analysis was used to evaluate the correlation between DDX3X mRNA and disease severity scoring systems (APACHE II and SOFA scores). Results showed that DDX3X mRNA level was positively correlated with APACHE II and SOFA scores (*r* = 0.24, *P* = 0.03; *r* = 0.27, *P* = 0.016, respectively) (Figures 2(a) and 2(b)).

Recent researches indicated that DDX3X is a contributing factor that leads to cell death under stress [15], and the reduction of cell number caused by T cell death is one of the main pathological manifestations of sepsis [16]. Interestingly, Spearman correlation coefficient analysis showed that DDX3X mRNA was significantly negatively correlated with T cell number (*r* = −0.36, *P* = 0.0012) (Figure 2(c)). These results suggest that DDX3X mRNA may be related to the loss of T cells.

### 3.4. Diagnostic Value of DDX3X mRNA in Sepsis Mortality

**Figure 3:**
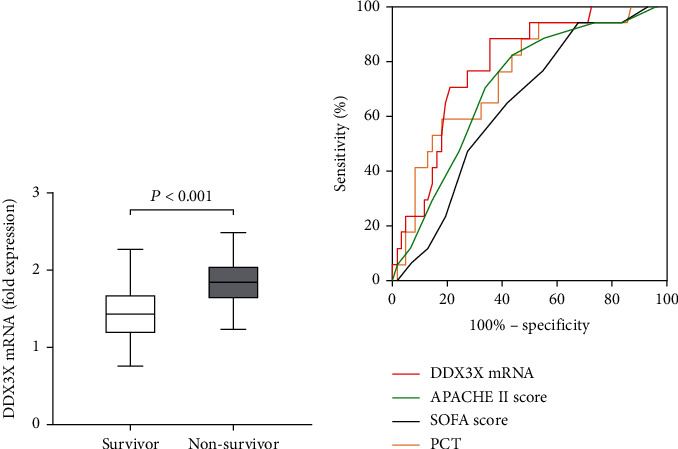
(a) The expression level of DDX3X mRNA in nonsurvivors was higher than that in the sepsis patients (1.84 ± 0.31 vs. 1.45 ± 0.37, *P* < 0.001). (b) ROC curves of DDX3X mRNA, SOFA score, APACHE II score, and PCT for predicting 28-day mortality in patients with sepsis and septic sepsis.

The level of DDX3X mRNA was higher in nonsurvivors as compared with survivors (Figure 3(a)). To evaluate the usefulness of DDX3X mRNA expression level in predicting 28-day mortality risk, we compared the predictive usefulness of DDX3X mRNA expression level, PCT, APACHE II, and SOFA in patients with sepsis by using receiver operating characteristic (ROC) curves. ROC curves of DDX3X mRNA, PCT, SOFA, and APACHE II were constructed according to statistically significant differences, and areas under the curves (AUCs) were calculated. The AUC of PCT, SOFA, and APACHE were 0.75 (95% CI, 0.62-0.87), 0.64 (95% CI, 0.51-0.77), and 0.71 (95% CI, 0.58-0.84), respectively. DDX3X mRNA was better than other indicators with AUC 0.79 (95% CI, 0.68-0.90) (Table 2 and Figure 3(b)).

**Figure 4:**
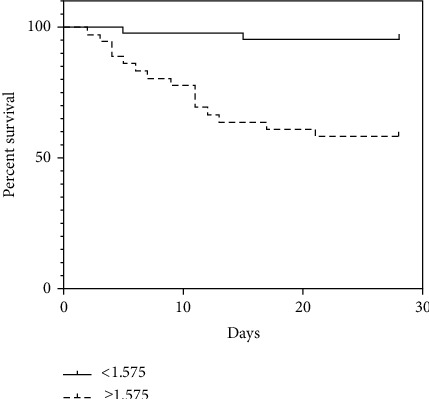
Kaplan-Meier survival analysis showed that the 28-day mortality of patients with level of DDX3X mRNA ≥ 1.575 was higher than that of patients with level of DDX3X mRNA < 1.575.

The best cutoff value of the DDX3X mRNA expression in T cells for predicting 28-day mortality in patients was 1.575 (sensitivity 88.24% and specificity 64.52%). Notably, Kaplan-Meier survival curves showed that patients with DDX3X mRNA higher than 1.575 had a higher risk of death than other patients (Log-rank test < 0.001) (Figure 4). This suggests that high mortality in sepsis patients is associated with high expression of DDX3X mRNA in T cells.

In multivariate analysis, the logistic regression model was adjusted for age, gender, lymphocyte count, PCT concentrations, the APACHE II score, SOFA score, and DDX3X mRNA in T cells to determine the relationship of 28-day mortality in patients with sepsis and septic shock. After univariate analysis, the PCT concentrations, the APACHE II, and DDX3X mRNA in T cells were included in the final model (*P* < 0.05). Multivariate logistic regression analysis showed that DDX3X mRNA on T cell is associated with the 28-day mortality with a hazard ratio of 9.540 (95% CI, 2.452-37.108) (Table 3).

## 4. Discussion

An imbalance between proinflammatory processes and immunosuppression is thought to contribute to sepsis, which is a fatal disease [17]. Some sepsis survivors who suffer from immune disorders can progress to PICS, leading to prolonged recovery after the acute phase inflammatory insult [7]. This state of sepsis is considered to occur through varieties of internal and external factors, including a lack of adaptive immune cell reactivity, dysfunction, or loss of T cells and B cells [7, 16]. Lymphocyte immunosuppression and dysfunction are key factors that contribute to the development of chronic immune disbalance and PICS [7]. Unfortunately, novel therapies targeted new biomarkers that may be potential to identify patients at high risk of infection and sepsis are still lacking. DDX3X, a member of the highly conserved DEAD-box RNA helicase family, have been demonstrated to be involved in the pathological processes of infection, immunity, and cell survival [12, 15, 18, 19]. In recent years, several studies have also found that DDX3X was an important regulator of cell death, including apoptosis and pyroptosis which are involved in sepsis [12, 15, 16, 20, 21]. However, it remains unclear whether DDX3X is involved in the pathological process of sepsis.

In this study, the level of DDX3X mRNA increased in T cells in patients with sepsis. The variation was not only correlated with the severity of sepsis but also associated with the 28-day mortality. Additionally, we found that DDX3X expression in T cells was negatively correlated with the cell number, suggesting that DDX3X may be involved in lymphocyte depletion and immunosuppression in sepsis. As far as we are aware, this is the first report of DDX3X expression in adaptive immune cells of sepsis, which may contribute to the assessment of disease severity and prognosis.

Sepsis is characterized by the coexistence of proinflammatory and anti-inflammatory reactions, along with abnormal phenotype or dysfunction of immune cells [22]. Therefore, investigation on the mechanism of immune imbalance is crucial for developing molecule-based therapies to prevent organ dysfunction and improve sepsis outcomes [2, 3]. Overwhelming studies have shown that T cell loss caused by apoptosis was not only one of the important pathological features in sepsis but also played a vital role in immunosuppression in sepsis [4, 5]. DDX3X serves as an important checkpoint of cell death, and it was initially found to be overexpressed in a variety of invasive cancers and associated with poor clinical prognosis [8, 9].

DDX3X plays an important role in immune regulation of T cells. For many years, DDX3X-specific T cells were considered to mediate graft versus leukemia effects as well as eradicated leukemia stem cells [23]. Previous research has also established that DDX3X-primed CD4(+) T cells produced CD133(+) tumor-specific IFN-*γ* and IL-17 and mediated potent antitumor therapeutic efficacy [18]. However, the role of DDX3X in regulating adaptive immune response in sepsis inflammation has not as yet been fully understood.

In view of these findings, we speculated that the expression level of DDX3X in T cells of sepsis patients may be different from that of healthy people, thus providing a potential biomarker for the diagnosis of sepsis. We therefore conducted this clinical trial and concluded that DDX3X mRNA expression level was significantly higher in patients with sepsis and septic shock than that in healthy volunteers and proved that DDX3X was associated with poor prognosis in sepsis.

By providing a clinical model, this study offers a novel understanding of DDX3X mRNA level in T cells significantly negatively correlated with T cell count in septic patients. As far as we are concern, reduction in the number of T cells is one of the critical causes of immunosuppression in sepsis [17]. Previous studies have implicated that the number of CD4+ T cells was one of the effective predictors of poor prognosis in sepsis [4]. Moreover, the depletion of T cells has always been considered as a contributor to apoptosis in sepsis [16]. Literature has confirmed that high level of DDX3X in p53 cells broadly activated external apoptotic pathways after DNA damage [12]. DDX3X regulates cell survival and apoptosis through the accumulation of p53 in blastocysts during early embryonic development in mice. In view of these findings, we speculated that the upregulation of DDX3X may be involved in the survival of T cells and contributes to T cell loss in sepsis. However, this hypothesis has not been fully explored and requires further investigation.

It is unclear to confirm reliable biomarkers to detect early clinical outcomes in patients with sepsis from the current findings. Up to now, the APACHE II score and SOFA score are still widely used to evaluate the severity of sepsis patients [24, 25]. In our cohort study, the level of DDX3X mRNA in T cells was significantly elevated in nonsurvivors compared with that in survivors, demonstrating its positive correlation with disease severity (APACHE II and SOFA scores).

PCT, SOFA, and APACHE II score have been widely used to facilitate sepsis diagnosis; however, their diagnostic and prognostic values are still limited [24, 26, 27, 28]. Emerging biomarkers are therefore in necessity for the prompt diagnosis of sepsis and prediction of outcomes. In our study, DDX3X mRNA, PCT, SOFA, and APACHE II score were compared by ROC curve to evaluate the effectiveness of DDX3X in predicting 28-day mortality in patients. ROC curve showed that DDX3X mRNA had a higher AUC than PCT, SOFA, and APACHE II score. DDX3X mRNA had the same sensitivity as PCT, but its specificity was higher than other parameters, suggesting that DDX3X mRNA could be used as a diagnostic marker of 28-day mortality in patients with sepsis. ROC curve and Cox hazard ratio model were used to evaluate the effect of DDX3X on mortality in septic patients. The results showed that the increased level of DDX3X mRNA (≥1.575) was associated with a significant increase in mortality. Therefore, the level of DDX3X mRNA on T cell may be a potential candidate for predicting 28-day mortality in sepsis.

Study limitations must be addressed. Firstly, it was a single-center study with a small sample size. The results of the current study need to be validated in a large, multicenter study. Secondly, we did not compare the expression differences of DDX3X between the septic and septic shock groups due to the relatively small number. Finally, this study was only an observational study and further studies are required to clarify the molecular mechanisms responsible for our findings.

## 5. Conclusion

In summary, this pilot study found that levels of DDX3X mRNA were significantly elevated in T lymphocytes of patients with sepsis and septic sepsis. In addition, DDX3X mRNA may be a potential biomarker for predicting the risk of death in patients with sepsis.

## Figures and Tables

**Table 1:** Baseline characteristics of the study population.

Variable	Sepsis (*n* = 52)	Septic shock (*n* = 27)	*P*
Gender (male), % (*n*)	59.62 (31)	70.37 (19)	0.4616
Age (year)	51.50 (43.00-60.50)	55.00 (47.00-63.00)	0.6615
APACHE II score	16.5 (15.00-19.00)	21.00 (18.00-22.00)	0.0024
SOFA score	6.00 (5.00-8.00)	9.00 (7.00-10.00)	0.0184
Underlying disease, % (*n*)			
Hypertension	15.38 (8)	18.52 (5)	0.7554
Diabetes	9.62 (5)	14.81 (4)	0.4825
Infection site, % (*n*)			0.6389
Respiratory	82.69 (43)	70.37 (19)	
Abdomen	5.77 (3)	11.11 (3)	
Blood	7.69 (4)	11.11 (3)	
Others	3.85 (2)	7.41 (2)	
Laboratory parameters			
WBC (×10^9^)	14.66 (11.77-18.28)	16.37 (14.02-18.72)	0.0936
LC (×10^9^)	0.47 (0.33-0.53)	0.39 (0.35-0.47)	0.1809
PCT (pg/mL)	3.66 (2.87-5.70)	4.63 (3.71-6.81)	0.0203
28-day mortality, % (*n*)	13.46 (7)	37.04 (10)	0.0219

Values are presented as median (95% confidence interval (CI)) or percentage (%). APACHE: acute physiology and chronic health evaluation; SOFA: sequential organ failure assessment; LC: lymphocyte count; WBC: white blood cells; PCT: procalcitonin.

**Table 2:** Areas under the ROC curves for predicting 28-day mortality in septic patients.

Parameter	AUC	Cutoff value	Sensitivity (%)	Specificity (%)	95% CI	*P*
DDX3X mRNA	0.79	1.575	88.24	64.52	0.68-0.90	0.0003
APACHE II score	0.71	17.50	82.35	56.45	0.58-0.84	0.0081
SOFA score	0.64	7.50	64.71	58.06	0.51-0.77	0.0795
PCT	0.75	3.64	88.24	52.23	0.62-0.87	0.0018

SOFA: sequential organ failure assessment; APACHE: acute physiology and chronic health evaluation; PCT: procalcitonin.

**Table 3:** Cox proportional hazard model of DDX3X mRNA and 28-day mortality.

	Univariate analysis			Multivariate analysis		
Variable	HR	95% CI	*P*	HR	95% CI	*P*
Sex (age)	0.799	0.304-2.098	0.651			
Age (year)	1.025	0.985-1.068	0.226			
APACHE II score	1.236	1.051-1.454	0.011	1.185	1.004-1.399	0.044
SOFA	1.158	0.962-1.395	0.121			
Lymphocyte count	0.052	0.001-3.864	0.178			
DDX3X mRNA	10.149	2.848-36.172	0.000	9.540	2.452-37.108	0.001
PCT	1.388	1.118-1.724	0.003	1.503	1.172-1.928	0.001

APACHE: acute physiology and chronic health evaluation; SOFA: sequential organ failure assessment; PCT: procalcitonin.

## Data Availability

The data used to support the findings of this study are available from the corresponding author upon request.
